# An Unexpected Regulatory Sequence from Rho-Related GTPase6 Confers Fiber-Specific Expression in Upland Cotton

**DOI:** 10.3390/ijms23031087

**Published:** 2022-01-19

**Authors:** Baoxia Li, Liuqin Zhang, Jing Xi, Lei Hou, Xingxian Fu, Yan Pei, Mi Zhang

**Affiliations:** 1 Biotechnology Research Center, Southwest University, No. 2 Tiansheng Road, Beibei, Chongqing 400715, China; libaoxia5854@163.com (B.L.); zhangliuqin0715@163.com (L.Z.); 15700351038@163.com (J.X.); houlei@swu.edu.cn (L.H.); fxx19950514@163.com (X.F.); peiyan3@swu.edu.cn (Y.P.); 2 Chongqing Key Laboratory of Plant Resource Conservation and Germplasm Innovation, Southwest University, Chongqing 400715, China; 3 Academy of Agricultural Sciences, Southwest University, Chongqing 400715, China

**Keywords:** fiber-specific promoter, cotton fiber, *GhROP6* promoter

## Abstract

Cotton fibers, single seed trichomes derived from ovule epidermal cells, are the major source of global textile fibers. Fiber-specific promoters are desirable to study gene function and to modify fiber properties during fiber development. Here, we revealed that *Rho-related GTPase6 (GhROP6)* was expressed preferentially in developing fibers. A 1240 bp regulatory region of *GhROP6*, which contains a short upstream regulatory sequence, the first exon, and the partial first intron, was unexpectedly isolated and introduced into transgenic cotton for analyzing promoter activity. The promoter of *GhROP6* (*proChROP6*) conferred a specific expression in ovule surface, but not in the other floral organs and vegetative tissues. Reverse transcription PCR analysis indicated that *proGhROP6* directed full-length transcription of the fused *ß-glucuronidase* (*GUS*) gene. Further investigation of GUS staining showed that *proChROP6* regulated gene expression in fibers and ovule epidermis from fiber initiation to cell elongation stages. The preferential activity was enriched in fiber cells after anthesis and reached to peak on flowering days. By comparison, *proGhROP6* was a mild promoter with approximately one-twenty-fifth of the strength of the constitutive promoter *CaMV35S*. The promoter responded to high-dosage treatments of auxin, gibberellin and salicylic acid and slightly reduced GUS activity under the in vitro treatment. Collectively, our data suggest that the *GhROP6* promoter has excellent activity in initiating fibers and has potential for bioengineering of cotton fibers.

## 1. Introduction

Cotton is a commercial crop providing the most extensively natural fibers used in global textile industries. Currently, genetic engineering has become a powerful approach to benefit cotton production, where transgenic cotton crops account for over 70% [1]. For targeted improvement of fiber properties, it is still a great challenge to allow desirable gene expression in specific tissues and time windows because some modifications, which promote fiber development, would not be favorable to the other tissues or plant growth. For example, improper manipulation of auxin biosynthetic gene *iaaM* results in abnormalities of the transgenic cottons, although auxin is known to be a vital plant hormone to increase fiber production [2]. In addition, a precise regulation of gene expression in fibers can also facilitate the study of gene expression and function without any systematic influences. Unlike generalized transformation of plants for insect or disease resistance, this must be tissue specific to the surface of developing cotton seeds and time specific to cellular stages. Thus, it is of great importance to isolate and characterize fiber-specific promoters in cotton.

Cotton fibers are differentiated ovule epidermal cells, and their development consists of five continuous stages: initiation, elongation, transition, secondary cell wall synthesis and maturation [3]. Thus far, a series of fiber-related promoters have been characterized with various activities. A part of them is the promoters with activity in fibers from the elongation to the secondary cell wall synthesis stages. *E6* and *FbL2A* are two promoters isolated due to the abundant transcription in cotton fibers [4,5]. *E6* and *FbL2A* show satisfactory specificity in fibers where their activity strengths are roughly equal to one-ninth and one-third, respectively, of that of the constitutive promoter *CaMV35S* [5]. Another promoter of lipase/hydrolase gene (*GhGDSL*) also displays fiber-specific activity mainly in the secondary cell wall synthesis stage [6]. Similar activity behavior is observed in promoters of chitinase-like protein (GhCTL) and TCP transcriptional factor (GbTCP), except for some additional activity in anthers, xylems or young cotyledons and roots [7,8]. In addition, the regulatory sequences of *RAC13*, *CelA1* and *LTP* also have activity in fibers of the elongation and secondary cell wall synthesis stages [9].

The other part of cotton fiber-related promoters is mainly active in fibers of the initiation and elongation stages. The promoter of a cotton expansion GbEXP2 is mainly active in elongating fibers, rather than in initiating fibers, but also has activity in the other expanding tissues, such as seed coats, young cotyledons, and hypocotyls [10]. Microtubules and microfilaments have been implicated in cotton fiber development. The promoter of ß-tubulin GhTUB1 is active in developing fibers but also in some other tissues, including pollens, ovaries, styles and cotyledons [11]. This similar activity is also reported in the promotor of ACTIN gene *GhACT1* [12]. Similar to other trichome cells, many transcriptional factors are involved in the regulation of cotton fiber initiation and elongation, and their promoters have thus been characterized. Promoters of RD22-like1 (RDL1), R2R3 MYB factors (GhMYB25 and GhMYB25-like), and HD-ZIP factor (GhHD-1) exhibit typical trichome-specific activity not only in developing fibers but also in other trichome cells [13,14,15,16]. Some extra activity in anthers, pollen grains, xylem vessels or roots is also observed in promoters of *GhMYB25*, *GhMYB25-Like* and *GhHD-1* [14,15,16]. In addition, another reported promoter of R2R3 MYB factor GhMYB109 displays strict fiber-specific behavior in the initiation stage [17]. The specificity is also seen in another promoter of a fiber-specific protease GhSCFP, and the activity spans from 0 to 36 DPA [18]. GbPDF1 is a PROTODERMAL FACTOR1 regulating cotton fiber initiation, and its promoter is predominantly active in ovule epidermis and developing fibers but also in some reproductive tissues and young seedlings [19].

In addition to the cotton-originated fibers, promoters from other plant species, such as *FBP7* (from *Petunia*) and *BAN* (from *Arabidopsis*), have been characterized with fiber-related activity [2]. The necessity of manipulating genes properly in improvement of fiber traits is also signified in the study [2]. Currently, by use of high-throughput technology, a great number of genes that are preferentially expressed in fibers have been identified. However, regulatory regions that can target gene transcription to fiber cells are still limited, especially ones with a detailed activity profile. This impedes our progress in understanding the mechanism of fiber development as well as in improving cotton fiber properties.

Rho-related GTPase 6 (GhROP6), a homolog of Arabidopsis ROP6, has a higher expression in fiber initials in contrast to non-fiber cells in the ovule epidermis in upland cotton (*Gossypium hirsutum* L.) [20]. Both GhROP6 copies from A- and D-subgenome exhibit a fiber-preferential expression due to the RNA-seq data [21]. Hence, in this study, we isolated a promoter sequence of GhROP6 and analyzed the activity in cotton by fusion with β-glucuronidase (GUS) reporter gene. Our data clearly indicate that the proGhROP6 is a fiber-specific promoter with preferential activity in the fiber initiation stage.

## 2. Results

### 2.1. GhROP6 Is Preferentially Expressed in Fiber Cells

**Figure 1:**
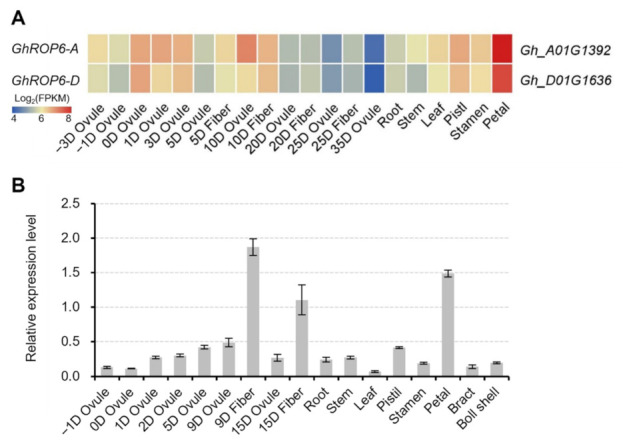
Expression profile of *GhROP6* in cotton tissues. (**A**) Heatmap of expression pattern of *GhROP6* copies. *GhROP6-A* (Gh_A01G1392) and *GhROP6-D* (Gh_D01G1636) are two copies of *GhROP6* in A- and D-subgenome of upland cotton. The heatmap was generated from the released data [21]. **(B**) Transcription level of *GhROP6* (in arbitrary units) normalized to that of *GhHIS3*. Error bars represent standard deviation of three technical repeats. Samples at −3 to 3 DPA (**A**) or −1 to 5 DPA (**B**) were fiber-bearing ovules.

The expression pattern of GhROP6 in cotton tissues was first examined according to the previous RNA-seq data [21]. Two copies (Gh_A01G1392 and Gh_D01G1636) of *GhROP6* showed a preferential expression in developing ovules and fibers apart from the highest transcription in petals (Figure 1A). Moreover, both, except for *GhROP6-A* at 10 DPA, showed a considerably higher expression in fiber tissues than in ovule tissues of 5 to 25 DPA (Figure 1A). The expression profile of *GhROP6* was further confirmed by quantitative RT-PCR (Figure 1B). Similarly, *GhROP6* was highly expressed in separated fibers and petals, although the transcript was detected in all tested tissues. The expression in 9-DPA fibers was the highest one, versus that in petals and in 15-DPA fibers. In addition, the expression in fibers was roughly three-fold higher than the separated ovule at 9 and 15 DPA. These data suggest that *GhROP6* is preferentially expressed in fibers of the early developmental stage.

### 2.2. Molecular Characterization of proGhROP6::GUS Transgenic Cotton

**Figure 2:**
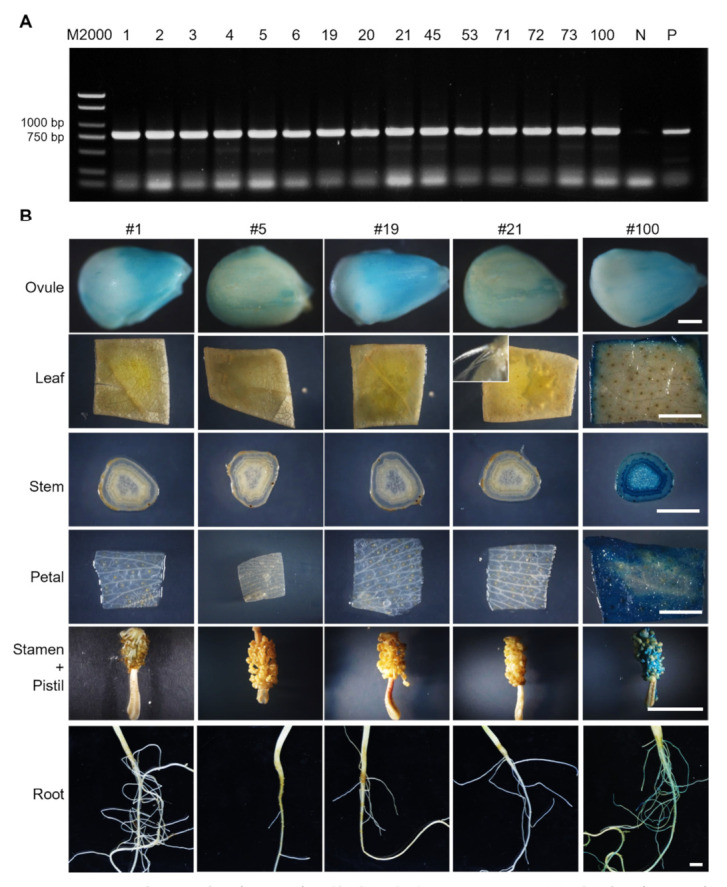
Identification of *proGhROP6::GUS* transgenic cotton. (**A**) PCR identification of introduction of selection gene *NPTII*. Number of transformants is shown in each lane. N represents the negative control. P represents the positive control of a *NPTII*-containing plasmid. (**B**) GUS staining in ovules, leaves, stems, petals, stamens, pistils, and roots. Floral organs were harvested at 0 DPA. The inset shows leaf trichomes. Scale bars represent 500 µm (ovule images) or 5 mm (the others).

A 1240 bp upstream regulatory sequence (Supplementary Materials, Figure S1), which contained a 478 bp region from the start codon ATG and the first exon (99 bp) and the partial first intron (662 bp) of *GhROP6-D*, was unexpectedly isolated from *Gossypium hirsutum* and fused with the *β-glucuronidase* (*GUS*) reporter gene to investigate promoter activity. In total, 15 transgenic cotton plants were obtained and confirmed by detection of the *NPTII* (kanamycin resistance) gene (Figure 2A) in the genome. GUS activity was then examined in 0-DPA ovules of these transgenic cottons. Eight plants showed visible staining in the ovule surface, and five (#1, #5, #19, #21 and #100) of them were further selected to analyze GUS activity in the other tissues (Figure 2B). Except for transformant #100 that showed strong staining in all tested tissues, all transformants showed no discernable activity in leaves (even in leaf trichomes), stems, petal, stamens, pistils and roots (Figure 2B). The absent activity in petals could be attributed to exclusion of the corresponding tissue-specific elements in the *GhROP6* promoter. These results indicate that the *proGhROP6* has strict activity in developing ovules.

### 2.3. The proGhROP6 Drives Normal Transcription of GUS Reporter Gene

**Figure 3:**
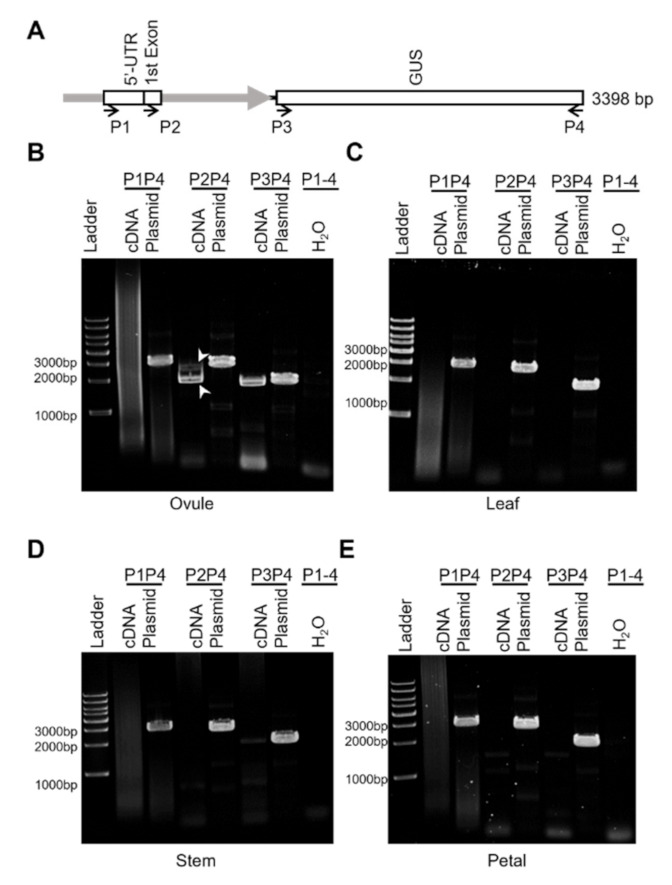
Transcription analysis of *proGhROP6::GUS* in the transgenic cotton. (**A**) Diagram of *proGhROP6::GUS* and primer location. *proGhROP6* contains the predicted 5′-UTR, the first exon, and part of the first intron of *GhROP6*. P1 to P4, primers for RT-PCR analysis; Plasmid, positive control using the plasmid as the template; H_2_O, negative control using the distilled water as the template. (**B**–**E**) RT-PCR analysis of *proGhROP6::GUS* transcripts. cDNA was synthesized using RNA extracted from 2-DPA ovules (**B**), leaves (**C**), stems (**D**) and petals (**E**). Arrowheads indicate the two transcripts of *proGhROP6::GUS* during RNA processing.

The inclusion of the first exon and the partial first intron of GhROP6 in the proGhROP6 sequence raised a concern about aberrant mRNA splicing of the target gene placed downstream. We thus analyzed three possible transcripts, starting from the predicted 5’-UTR, the first exon of GhROP6, and the start codon of GUS with selective primer pairs (Figure 3A) in tissues of *proGhROP6::GUS* transgenic cotton. *GUS* transcript was detected in cDNA extracted from 2-DPA ovules, while two bands were amplified out with the upper stream primer locating at the first exon of *GhROP6* (Figure 3B). We sequenced them, and the results showed that the long one was the predicted transcript and the short one had a splicing in the partial intron region of the *proGhROP6* promoter (Supplementary Materials, Figure S1). No other splicing was found in the coding region of *GUS*. We did not detect any specific amplicon with the primer located at the 5’-UTR (Figure 3B). A full-length transcriptome analysis was then performed to estimate GUS-related transcripts in 2-DPA ovules of the transgenic cotton. Consistent to the RT-PCR analysis, only two types of the transcripts were detected, of which one was the full-length transcript containing the first exon and the partial first intron involved in the proGhROP6, and the other was short because the partial first intron was removed (Supplementary Materials, Figure S1). We also analyzed *proGhROP6::GUS* transcription in leaves, stems and petals of the transgenic cotton, where GUS staining was invisible. No other specific amplicons, except for faint transcription of *GUS* in the stem, were detected with all three primer pairs in these tissues (Figure 3B–E). These results suggest that the detected GUS activity on ovule surface is due to the correct transcription of *GUS* gene, and the *proGhROP6* does not direct any abnormal processing of *GUS* in cotton tissues.

### 2.4. Activity of the proGhROP6 in Developing Ovules

**Figure 4:**
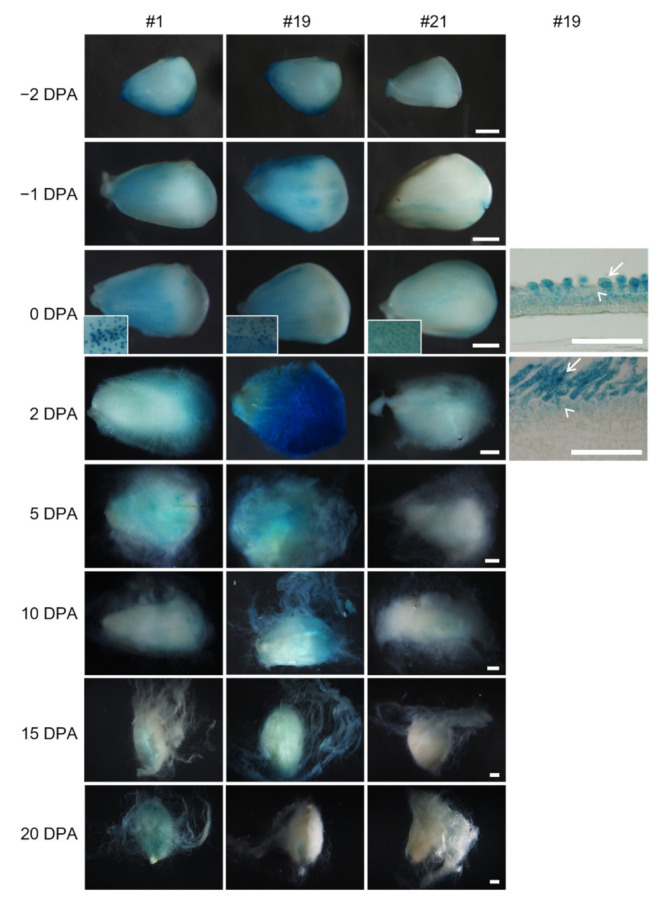
GUS activity in developing ovules of *proGhRop6::GUS* transgenic cotton. Gus staining in ovules of transformant #19 were further observed through paraffin-embedded sections. Arrows indicate fiber cells, and arrowheads indicate non-fiber cells in ovule epidermis. Scale bars represent 500 µm (ovule images) or 50 µm (sections).

Next, we analyzed activity of the *GhROP6* promoter in developing ovules of transformants #1, #19 and #21. All three transformants showed a similar GUS expression pattern (Figure 4). GUS staining on the ovule surface was of peak value at 0 or 2 DPA and then decreased after 5 DPA. This tendency was obvious in transformant #19 and #21, where the staining was weak at 10 to 20 DPA. The GUS activity was mainly enriched in ovule epidermal cells before anthesis. However, dotted blue staining on the ovule surface (insets in Figure 4) indicated stronger activity in fiber cells after anthesis, which was further confirmed in ovule sections. Thus, the promoter of *GhROP6* is mainly active in fiber cells of the initiation stage.

### 2.5. The proGhROP6 Is a Weak Promoter Active in Fiber Initiation Stage

**Figure 5:**
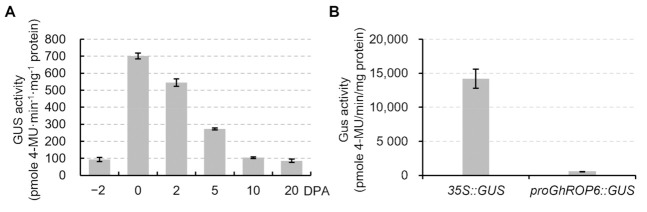
Strength of GUS activity in *proGhROP6::GUS* ovules. (**A**) GUS activity in developing ovules. Samples from −2 to 2 DPA were fiber-bearing ovules, and those from 5 to 20 DPA were separated fibers. (**B**) Comparison of GUS activity in 0 DPA ovules between *proGhROP6::GUS* and *35S::GUS* cottons. Error bars represent standard deviations of three biological replicates.

In order to verify strength of the *proGhROP6* activity, we quantified GUS activity in developing ovules (−2 to 2 DPA) and fibers (after 2 DPA) of *proGhROP6::GUS* transformant #1 (Figure 5A). The GUS activity reached the highest level at 0 DPA and then decreased to a base level at 10 DPA, in which the value was similar to that at −2 and 20 DPA. Approximately, the 0 DPA value was seven times as high as the base level. We compared activity of the *proGhROP6* with the constitutive promoter *CaMV35S (35S)*. GUS activity of *proGhROP6::GUS* ovules was nearly one-twenty-fifth of that in *35S::GUS* ovules (Figure 5B). These data suggest that *proGhROP6* is a mild promoter active in the fiber initiation stage.

### 2.6. Responses of the GhROP6 Promoter to Plant Hormones

**Figure 6:**
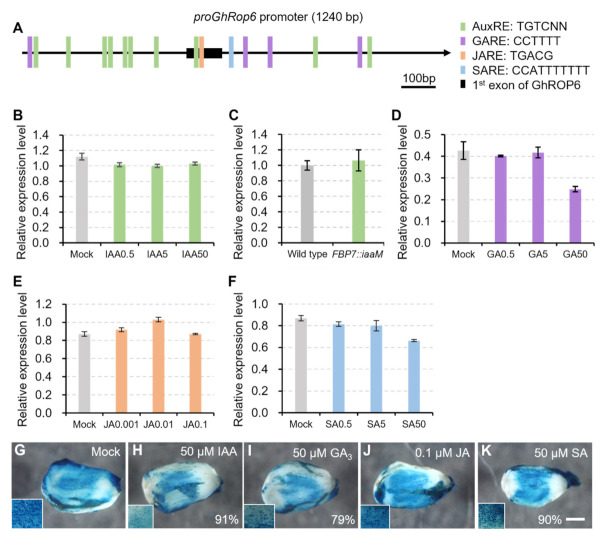
Activity of GhROP6 promoter in response to different plant hormones. (**A**) Response elements for different plant hormones in the *GhROP6* promoter region. (**B**) Transcription level of *GhROP6* in wild-type ovules treated with different concentration of IAA. (**C**) Transcription level of *GhROP6* in *FBP7::iaaM* and wild-type ovules. (**D**) Transcription level of *GhROP6* in wild-type ovules treated with different concentration of GA_3_. (**E**) Transcription level of *GhROP6* in wild-type ovules treated with different concentration of MeJA. (**F**) Transcription level of *GhROP6* in wild-type ovules treated with different concentration of SA. Transcription level (in arbitrary units) was normalized to that of *GhHIS3*. Error bars represent standard deviation of three repeats. (**G**–**K**) GUS staining in *proGhROP6::GUS* transgenic ovules after treatment with different plant hormones. Over twenty ovules of transformant #1 were harvested for each treatment. Percentage of ovules with reduced GUS staining are shown. GUS staining of ovules in the lower concentrations were indistinguishable from the control. Insets show enlargement of ovule surface. The scale bars represent 500 μm. Ovules at 0 DPA, except for those in (**C**), were treated with different plant hormones for 6 h.

A series of cis-acting regulatory elements in response to plant hormones auxin, gibberellin, jasmonate, and salicylic acid were detected (Figure 6A) using the database PlantCARE (http://bioinformatics.psb.ugent.be/webtools/plantcare/html/ (accessed on 26 October 2021)) or the reported motifs [22,23]. This implied that this *proGhROP6* might be responsive to these hormones. To test it, wild-type ovules were treated with these plant hormones to analyze expression of *GhROP6*. *GhROP6* transcription was slightly decreased in the presence of IAA at three concentrations (0.5, 5.0 and 50 μM), but the responses were not dose-dependent (Figure 6B). We compared the *GhROP6* transcription to that in *FBP7::iaaM* ovules, in which auxin level had been genetically increased in the ovule epidermis [2]. No discernable change was detected as compared to wild-type ovules (Figure 6C), suggesting that the induced decrease in *GhROP6* expression by auxin is limited. Decreased expression of *GhROP6* was also detected in gibberellin acid (GA_3_)-treated ovules, but only at the high concentration of 50 μM (Figure 6D). Similar results were also detected in ovules treated with salicylic acid (Figure 6F). In contrast, no trend of *GhROP6* expression was seen in methyl jasmonate (MeJA)-treated ovules (Figure 6E). We further confirmed the response of the *GhROP6* promoter using *proGhROP6::GUS* transgenic ovules. Consistent to the result of *GhROP6* transcription, GUS staining on ovules had no discernable changes when treated by the lower concentrations of the plant hormones. While at the highest concentration, a large portion (from 79% to 91%) of ovules with slightly decreased GUS staining were observed in the presence of IAA, GA_3_ or SA, and the GUS signal in MeJA treatment was undistinguishable compared with the control (Figure 6G–K). All these treatments did not change the enriched activity of *GhROP6* promoter in fiber cells (insets in Figure 6G–K). These results suggest that the *GhROP6* promoter is possibly responsive to auxin, gibberellin and salicylic acid at an extremely high concentration.

## 3. Discussion

Cotton fiber is the most valuable natural fiber source in the world. To study cell developmental processes or to improve fiber properties, it is of great importance to understand and genetically modify gene transcription involved in fiber development, temporally and spatially, for the desired effects on fiber development. In the present study, we provided a regulatory region from ROP GTPase GhROP6, which exhibited outstanding activity in fiber cells of thee initiation stage.

What distinguishes *proGhROP6* from the other fiber related promoters? Tens of fiber-related promoters have been characterized, among which only promoters of *GhMYB109* and *GhSCFP* show restricted tissue-specific activity in initiating cotton fibers. *GhMYB109* promoter has a peak level in 5 DPA fibers [17]. *GhSCFP* promoter is active in a long period from the beginning of fiber initiation until almost the end of secondary cell wall synthesis [18]. Moreover, the expression is too strong to finely control endogenous plant hormones in transgenic plants [2]. By contrast, activity of the *GhROP6* promoter was active in fiber cells and epidermis, especially for the epidermal cells before anthesis. It is advantageous for exploring function of genes involved in fiber differentiation because fibers are differentiated epidermal cells. Additionally, the activity spanned two fiber developmental stages—initiation and elongation, which was strikingly high when the fibers initiated. The comparatively mild activity of *proGhROP6* will enhance regulation of some growth factors, such as plant hormones, for which fine tuning of the endogenous level is required.

The involvement of the first exon and the partial first intron of GhROP6 in the *proGhROP6* caused no abnormal transcription of the downstream gene. Our results also indicate that the involvement may be unnecessary for the tissue-specific activity of the promoter because of the detection of the transcript starting from the first exon and the removal of the majority of the partial first intron in the transgenic cotton, where GUS staining was predominantly restricted to initiating fiber cells. Thus, the minimal regulatory sequence (the initial 478 bp sequence without the following exon and partial intron sequence) of the *proGhROP6* could be an option if there are any concerns about the additional transcriptional sequence at the 5’ end of the downstream gene.

The induced activity of the *proGhROP6* by plant hormones has little limitation on the use of this promoter. Our present study showed that the responses to auxin, gibberellin and salicylic acid were slight and were only detected in in vitro treatment at a high dosage. Auxin and gibberellin have been reported as two key plant hormones regulating the fiber initiation and elongation [2,24,25,26]. With auxin and GA_3_ as an example, the dosage is much higher than the optimum concentration to produce cotton fibers [27]. The unchanged expression of *GhROP6* in *FBP7::iaaM* ovules also suggests that the normal range of auxin in cotton fibers does not induce a decline in *proGhROP6* activity.

Combined with the similar expression patten of *proGhROP6::GUS* in multiple transgenic cottons, our data suggest that the *proGhROP6* is an effective regulatory region to modify gene transcription and fiber traits in cotton fibers of the initiation stage.

## 4. Materials and Methods

### 4.1. Plasmid Construction and Plant Materials

The 1240 bp upstream fragment of the star codon of *GhROP6* in the D-subgenome was amplified from upland cotton ‘Jimian 14’ with primers 5′-attaagcttcagaactttcttatttcactg-3′ and 5′-attggatccacccaagattcgcagtaatgaaagc-3′ and then fused with *β-glucuronidase* (*GUS*) reporter gene in binary vector pBI121 at sites *Hin*dIII and *Bam*HI. The construct was introduced into upland cotton ‘Jin668’ with the *Agrobacterium*-mediated method [28]. Kanamycin-resistant plantlets were screened out and further identified by amplification of selection gene *NPTII* (795 bp) with primers 5′-atgattgaacaagatggattgcacg-3′ and 5′-tcagaagaactcgtcaagaaggcga-3′. *FBP7::iaaM* cotton (Line 9) and the wild type were described in the previous study [2]. Transformants were grown in a greenhouse receiving natural daylight.

### 4.2. qPCR and RT-PCR Assays

RNA was extracted from ovules and fibers using an EASY spin plant RNA extraction kit (Aidlab, Beijing, China). Approximately 1 µg RNA was used to synthesize first-strand cDNA with a PrimeScript^TM^ RT Reagent kit with gDNA Eraser (TaKaRa, Kusatsu, Shiga, Japan). qPCR was performed on a CFX Connect™ Real-Time System (Bio-Rad, Hercules, CA, USA) with ChamQ™ Universal SYBR qPCR Master Mix (Vazyme, Nanjing, China), and the data were analyzed using the software CFX manager (Bio-rad, Hercules, CA, USA). The thermal cycling consisted of a pretreatment (94 °C, 3 min) followed by 40 amplification cycles (94 °C, 20 s; 56 °C, 20 s; 72 °C, 30 s). Gene-specific primers 5′-tcacaaaaggcctgctcgat-3′ and 5′-acagactacaaaccaaaaggagc-3′ were used for detecting transcription of *GhROP6*, and 5’-gaagcctcatcgataccgtc-3′ and 5′-ctaccactaccatcatggc-3′ for the reference gene *GhHIS3* (AF024716). Each test was confirmed by three individual runs (biological replicates) and data from one of them were used to determine expression. For detection of the possible transcripts caused by *proGhROP6::GUS* expression, four primers 5′-gtccaagagctgtctttcctttcc-3′ (P1), 5′-atgagtgcatcaaggttcatcaaatg-3′ (P2), 5′-atgttacgtcctgtagaaacccca-3′ (P3), and 5’-tcattgtttgcctccctgct-3′ (P4) were used for RT-PCR analysis. RNA was also sent to the BENAGEN company for a full-length transcriptome analysis.

### 4.3. Analysis of GUS Activity

Histochemical GUS staining was performed using a previous method [29]. Cotton tissues were stained in the dark at 37 °C for 12 h and then were washed and kept in 75% ethanol for observation. Images were captured on a V20 stereo-microscope imaging system (Zeiss, Oberkochen, Germany). Determination of GUS activity was performed using a previous method [25]. Fifty fresh ovules of each sample were ground to obtain the total protein for the assay.

### 4.4. Ovule Culture

Stock solutions of IAA and GA_3_ were prepared in distilled water [29]. Methyl jasmonate (Sigma, St. Louis, MO, USA) and sodium salicylate (Sigma, St. Louis, MO, USA) were dissolved in DMSO to prepare stock solutions of MeJA (10 mM) and SA (50 mM), respectively. Ovule culture was carried out as described before [29]. Ovule at 0 DPA were cultured on the BT medium with different plant hormones in the darkness for 6 h. Treatment with an equal volume of distilled water or DMSO was used as the parallel control. Then, those ovules were harvested for qPCR analysis or microscopic observation.

## Data Availability

The data presented in this study are available on request from the corresponding author.

## Supplementary Materials

The following supporting information can be downloaded at: https://www.mdpi.com/article/10.3390/ijms23031087/s1.
