# Supplementary material for: An Unexpected Regulatory Sequence from Rho-Related GTPase6 Confers Fiber-Specific Expression in Upland Cotton
Source: Int J Mol Sci. 2022 Jan 19;23(3):1087. doi: 10.3390/ijms23031087 (PMC8834676; doi:10.3390/ijms23031087)
Supplement: Supplementary file 1 [file ijms-23-01087-s001.zip › ijms-1559150-supplementary.pdf]

1 cagaactttc ttatttcact gttaatttcc ttttaattgc attttctgtc attcaatcat
61 tcttatcgaa tcattaaaat acattattat taaaaaaaca aataaatact taatttaaaa
121 ttttaaaaat atgtcatact tgaaataact caataagccc ttcaatgaac tttttaggta
181 taataataaa ataaataata aattatttat ggaaaatata ttgttgtaat gtaagcagat
241 agctgtccaa gagctgtctt tcctttccct aatgattctc tctctttctt tcgggcaatg
301 tctttgatgt cttccagttt tacttagtcc ttcatcttct tcttcatttc agtgtttttg
361 ggtttgtttt ttttcctact gttttggaca ataattgaag caagagataa ccttgggggc
421 ggcggcggag ggagaggaac atctctgaaa ttactttcac ttttgccggt gggagtaaaa
481 tgagtgcatc aaggttcatc aaatgtgtca ctgttggtga cggtgccgtc ggcaagactt
541 gcatgctcat ctcctacacc agcaatactt tccctactgt gagttccaaa aagaaagaaa
601 ctaacccatt tttttgttt ggtctctgag aaaatatggg gaatggaaaa ggaagtttgg
661 ctggccctta acgttttctt tacttggatt atcatggatt ttcttttctt tttctttccc
721 ttttgtttc ccctattttc ttggccacca agcagaccct acactggttt ctggtggtca
781 atgtaacttt ggtaaagttg ctttctttat tgggtttttt tttgtttctt tttcccattt
841 tgttgtactt gtctttggaa tcttgaattt ggggcatagt atctttctga ataatagaat
901 cattcttcgt aaattgtgtt caagtgagca atttcttagg gtttttttgct tcaaaaactt
961 gcagaaaatt tattgccttt ttgtggttta taccttctta tttcttctgt ttgtgttttg
1021 atgatgcatc atctaggata aagattcttt gtcttgtagg ttttcatttc aattagtgag
1081 ttaaggtcaa aagttaaaac tggaaacatt atatcctgct actcccctgt tttttgctgg
1141 aggagaaaga aaacaaattg ctaaatctga aactattgct ggtagaaagt ctcatttaga
1201 gatttcataa taggtgcttt cattactgcg aatcttgggt ggatccccgg gtggtcagtc
1261 ccttATG

nn AuxRE nn GARE nn JARE nn SARE nn Spacer nn Start code of GUS

nn Predicted 5'-UTR nn First exon nn Possibly removed sequence in RNA processing

Figure S1 Sequence information of the isolated GhROP6 promoter in the expression vector
